# Supplementary figures and images for: Dose-response analysis between hemoglobin A1c and risk of atrial fibrillation in patients with and without known diabetes
Source: PLoS One. 2020 Feb 18;15(2):e0227262. doi: 10.1371/journal.pone.0227262 (PMC7028260; doi:10.1371/journal.pone.0227262)

**
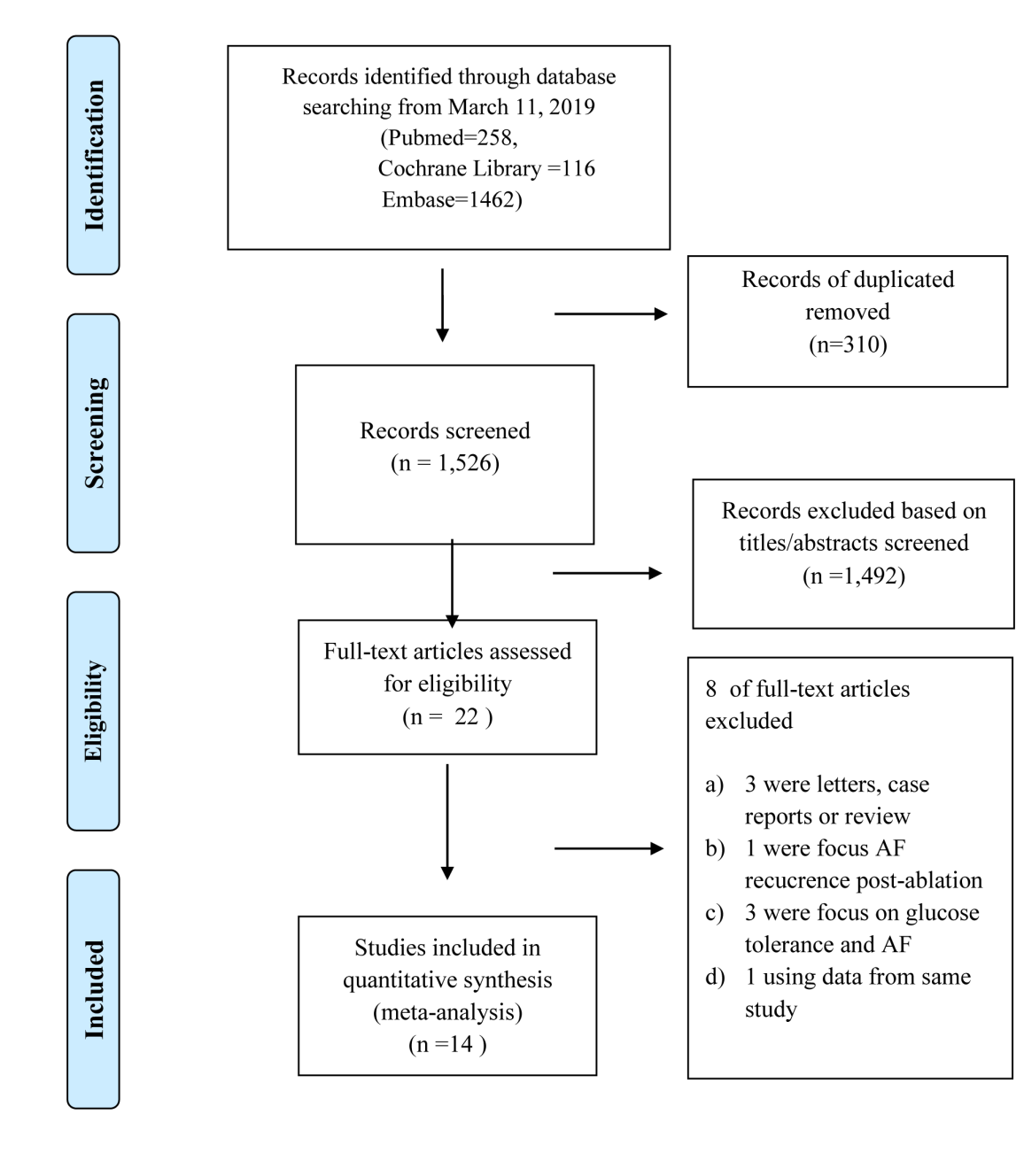
**

**Figure S1. Flowchart of study selection.**

Supplement: S1 Fig — (DOCX) [file pone.0227262.s001.docx]
