# Supplementary material for: Dose-response analysis between hemoglobin A1c and risk of atrial fibrillation in patients with and without known diabetes
Source: PLoS One. 2020 Feb 18;15(2):e0227262. doi: 10.1371/journal.pone.0227262 (PMC7028260; doi:10.1371/journal.pone.0227262)
Supplement: S2 Fig — (DOCX) [file pone.0227262.s002.docx]

**
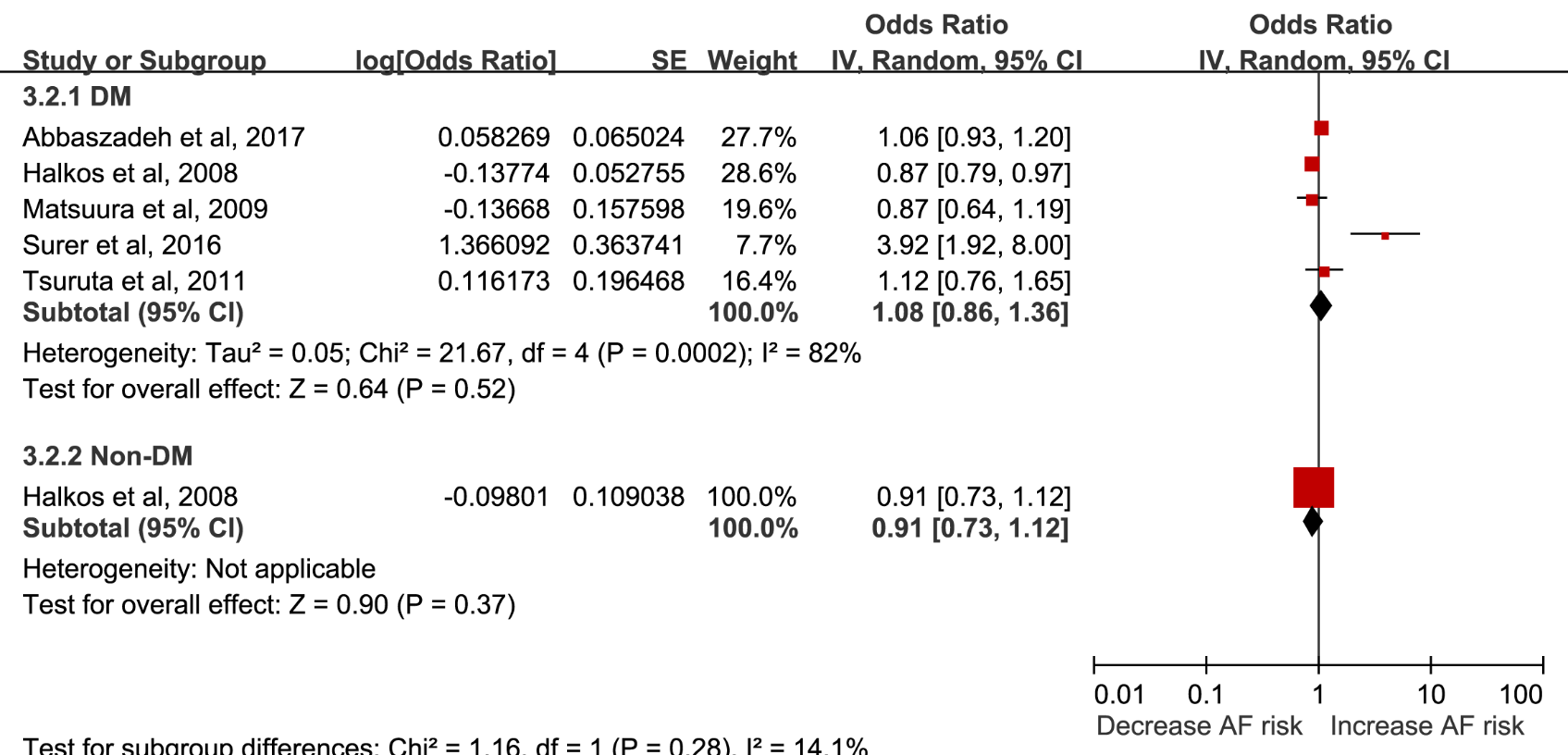
**

**Figure S2. Forest plot of HbA1c and atrial fibrillation incidence among diabetes and non-diabetes patients undergoing coronary artery bypass, per 1% increase.**
