# Supplementary material for: Dose-response analysis between hemoglobin A1c and risk of atrial fibrillation in patients with and without known diabetes
Source: PLoS One. 2020 Feb 18;15(2):e0227262. doi: 10.1371/journal.pone.0227262 (PMC7028260; doi:10.1371/journal.pone.0227262)
Supplement: S2 Table — (DOCX) [file pone.0227262.s004.docx]

**Table S2: Search strategy
PubMed (search result=258)**

#1 (atrial fibrillation [Title/Abstract]) OR atrial tachycardia [Title/Abstract] OR supraventricular tachycardia [Title/Abstract])

#2 ((((((((((((((glycated hemoglobin[Title/Abstract]) OR glycosylated hemoglobin[Title/Abstract]) OR glycosylated haemoglobin[Title/Abstract]) OR glycated haemoglobin[Title/Abstract]) OR hemoglobin A1[Title/Abstract]) OR hemoglobin A1c[Title/Abstract]) OR HbA1[Title/Abstract]) OR HbA1c[Title/Abstract]) OR Haemoglobin A1c[Title/Abstract]) OR Haemoglobin A1[Title/Abstract]) OR hemoglobin a, glycosylated[MeSH Terms]) OR A1c[Title/Abstract]) OR glycaemic control[Title/Abstract]) OR glycemic control[Title/Abstract]) OR A1[Title/Abstract]

#3 #1 AND #2

**Cochrane Library (search results=116)**

#1 (atrial fibrillation [Title/Abstract]) OR atrial tachycardia [Title/Abstract] OR supraventricular tachycardia [Title/Abstract])

#2 ((((((((((((((glycated hemoglobin[Title/Abstract]) OR glycosylated hemoglobin[Title/Abstract]) OR glycosylated haemoglobin[Title/Abstract]) OR glycated haemoglobin[Title/Abstract]) OR hemoglobin A1[Title/Abstract]) OR hemoglobin A1c[Title/Abstract]) OR HbA1[Title/Abstract]) OR HbA1c[Title/Abstract]) OR Haemoglobin A1c[Title/Abstract]) OR Haemoglobin A1[Title/Abstract]) OR hemoglobin a, glycosylated[MeSH Terms]) OR A1c[Title/Abstract]) OR glycaemic control[Title/Abstract]) OR glycemic control[Title/Abstract]) OR A1[Title/Abstract])

#3 #1 AND #2

**EMBASE Database(search results=1526)**

#1 atrial fibrillation:ab,ti AND ([article]/lim OR [article in press]/lim OR [conference abstract]/lim OR [conference paper]/lim)

#2 atrial tachycardia:ab,ti AND ([article]/lim OR [article in press]/lim OR [conference abstract]/lim OR [conference paper]/lim)

#3 supraventricular tachycardia:ab,ti AND ([article]/lim OR [article in press]/lim OR [conference abstract]/lim OR [conference paper]/lim)

#4 #1 OR #2 OR #3

#5 glycated AND hemoglobin:ab,ti AND ([article]/lim OR [article in press]/lim OR [conference abstract]/lim OR [conference paper]/lim)

#6 glycosylated AND hemoglobin:ab,ti AND ([article]/lim OR [article in press]/lim OR [conference abstract]/lim OR [conference paper]/lim)

#7 glycosylated AND haemoglobin:ab,ti AND ([article]/lim OR [article in press]/lim OR [conference abstract]/lim OR [conference paper]/lim)

#8 glycated AND haemoglobin:ab,ti AND ([article]/lim OR [article in press]/lim OR [conference abstract]/lim OR [conference paper]/lim)

#9 'hemoglobin'/exp OR hemoglobin AND a1:ab,ti AND ([article]/lim OR [article in press]/lim OR [conference abstract]/lim OR [conference paper]/lim)

#10 'hemoglobin'/exp OR hemoglobin AND a1c:ab,ti AND ([article]/lim OR [article in press]/lim OR [conference abstract]/lim OR [conference paper]/lim)

#11 hba1:ab,ti AND ([article]/lim OR [article in press]/lim OR [conference abstract]/lim OR [conference paper]/lim)

#12 hba1c:ab,ti AND ([article]/lim OR [article in press]/lim OR [conference abstract]/lim OR [conference paper]/lim)

#13 'haemoglobin'/exp OR haemoglobin AND a1c:ab,ti AND ([article]/lim OR [article in press]/lim OR [conference abstract]/lim OR [conference paper]/lim)

#14 'haemoglobin'/exp OR haemoglobin AND a1:ab,ti AND ([article]/lim OR [article in press]/lim OR [conference abstract]/lim OR [conference paper]/lim)

#15 a1c:ab,ti AND ([article]/lim OR [article in press]/lim OR [conference abstract]/lim OR [conference paper]/lim)

#16 glycaemic AND control:ab,ti AND ([article]/lim OR [article in press]/lim OR [conference abstract]/lim OR [conference paper]/lim)

#17 glycemic AND control:ab,ti AND ([article]/lim OR [article in press]/lim OR [conference abstract]/lim OR [conference paper]/lim)

#18 #5 OR #6 OR #7 OR #8 OR #9 OR #10 OR #11 OR #12 OR #13 OR #14 OR #15 OR #16 OR #17

#19 #4 AND # 18
