# Supplementary material for: Dose-response analysis between hemoglobin A1c and risk of atrial fibrillation in patients with and without known diabetes
Source: PLoS One. 2020 Feb 18;15(2):e0227262. doi: 10.1371/journal.pone.0227262 (PMC7028260; doi:10.1371/journal.pone.0227262)
Supplement: S3 Table — (DOCX) [file pone.0227262.s005.docx]

**Table S3**. Quality assessment of included studies

| Author  (Publication Year) | Newcastle-Ottawa Scale | | | | | | | | | |
| --- | --- | --- | --- | --- | --- | --- | --- | --- | --- | --- |
|  | Selection | | | Comparability | | | Outcome | | | Total |
|  | a | b | c | d | e | f | g | h | i |  |
| Halkos, 2008,  USA | 1 | 0 | 1 | 1 | 0 | 0 | 1 | 1 | 0 | 5 |
| Matsuura, 2009,  Japan | 1 | 1 | 1 | 1 | 1 | 1 | 0 | 0 | 0 | 6 |
| Dublin, 2010,  USA | 1 | 1 | 1 | 1 | 1 | 1 | 1 | 1 | 0 | 8 |
| Tsuruta, 2011,  Japan | 1 | 1 | 0 | 1 | 1 | 0 | 0 | 1 | 0 | 5 |
| Huxley, 2012,  USA | 1 | 1 | 1 | 1 | 1 | 1 | 1 | 1 | 0 | 8 |
| Kinoshita, 2012,  Japan | 1 | 1 | 1 | 1 | 1 | 1 | 1 | 0 | 0 | 7 |
| Iguchi, 2012,  Japan | 1 | 1 | 0 | 1 | 1 | 1 | 1 | 0 | 0 | 6 |
| Turgut, 2013,  Turkey | 1 | 1 | 1 | 0 | 1 | 1 | 1 | 0 | 0 | 6 |
| Latini,2013,  Italy | 1 | 1 | 1 | 1 | 1 | 1 | 1 | 1 | 0 | 7 |
| Sandhu, 2014,  Canada | 1 | 1 | 0 | 1 | 1 | 1 | 1 | 1 | 0 | 7 |
| Blasco, 2014,  Spain | 1 | 1 | 1 | 1 | 0 | 1 | 1 | 0 | 0 | 6 |
| Surer, 2016,  Turkey | 1 | 1 | 1 | 0 | 1 | 1 | 1 | 0 | 0 | 6 |
| Abbaszadeh, 2017,  Iran | 1 | 1 | 1 | 1 | 0 | 1 | 1 | 1 | 0 | 7 |
| Dahlqvist, 2017, Sweden | 1 | 1 | 1 | 1 | 1 | 1 | 1 | 1 | 0 | 8 |

1. Representativeness of the exposed cohort.
2. Selection of the non-exposed cohort.
3. Ascertainment of exposure.
4. Demonstration that the outcome of interest was not present at the start of the study.
5. Comparability of cohorts on the basis of the design or analysis (age).
6. Comparability of cohorts on the basis of the design or analysis (any other factor).
7. Assessment of outcome.
8. Was follow-up long enough for outcomes to occur (more than 1 years).
9. Adequacy of follow-up of cohorts.
